# Cutaneous Anaplastic T-Cell Lymphoma Mimicking a Small Abscess: A Case Report

**DOI:** 10.7759/cureus.64455

**Published:** 2024-07-13

**Authors:** Kanishka Goswami, Gurjot Singh, Aishmeet Singh, Shubam Trehan, Meet Popatbhai Kachhadia

**Affiliations:** 1 Internal Medicine, Maharaj Sawan Singh Charitable Hospital, Beas, IND

**Keywords:** mri gluteal region, brentuximab, cutaneous anaplastic t-cell lymphoma, chronic non-healing ulcer, non-healing ulcer

## Abstract

Non-healing ulcers display a noteworthy demonstrative challenge for clinicians. While often attributed to common causes like infections, these persistent wounds can occasionally mask a more sinister underlying condition: malignancy. This case report presents a 39-year-old Indian man with a non-healing ulcer on his right gluteal region. Despite initial treatments for a presumed bacterial infection, the ulcer persisted. Biopsy ultimately revealed a malignant neoplasm of possible hematopoietic origin, positive for CD30 and focally positive for CD45. Further investigations, including MRI, FNAC, and X-rays, were indicative of lymphoma.

Non-healing ulcers present a challenge due to diverse etiologies. A thorough understanding of potential causes, including infectious, vascular, autoimmune, and malignant etiologies, is crucial for navigating the diagnostic process. This case highlights the critical role of maintaining a broad differential diagnosis for non-healing ulcers and the importance of a biopsy in reaching a definitive diagnosis. Early recognition of malignancy in such cases is essential for optimal patient management. This case underscores the importance of considering malignancy in patients with persistent ulcers and performing biopsies for a definitive diagnosis. While the initial presentation mimicked an infectious process, the biopsy revealed a possible cutaneous anaplastic T-cell lymphoma. Further investigations are necessary to definitively classify the specific lymphoma subtype and guide further treatment decisions.

## Introduction

Non-healing ulcers represent a significant diagnostic conundrum in clinical practice. These persistent wounds can arise from a multitude of underlying etiologies, each requiring distinct treatment approaches. A thorough understanding of the potential causes is crucial for navigating this diagnostic labyrinth.

In the realm of infectious diseases, bacterial infections are a frequent culprit. *Staphylococcus aureus* and *Pseudomonas aeruginosa* are notorious for establishing persistent infections [1]. Fungal infections, such as those caused by *Candida* or *Mucormycosis* species, can also manifest as non-healing ulcers, especially in diabetic patients or those on prolonged antibiotic therapy [2]. Additionally, tuberculosis can present as a chronic ulcer, though less commonly [3]. An interferon-gamma release assay was performed in this case to rule out tuberculosis, with negative results.

Beyond infectious etiologies, vascular insufficiency, autoimmune disorders, and malignancies can also present with chronic ulcers [4-6]. Diabetic patients with compromised circulation or individuals with vasculitis can develop ulcers due to tissue ischemia. Similarly, chronic inflammatory bowel diseases like Crohn's disease can involve the skin [7,8], resulting in non-healing ulcers. Even malignancies like squamous cell carcinoma or, in rare instances, certain lymphomas or sarcomas, can mimic an ulcer [9-13].

This case report explores the diagnostic journey of a 39-year-old Indian male who developed a non-healing ulcer on his right gluteal region. The initial presentation mimicked a bacterial abscess, leading to treatment with antibiotics, incision, and drainage. However, the ulcer persisted, prompting further investigation. The workup in this case ultimately revealed a possible cutaneous anaplastic T-cell lymphoma, a rare form of cancer. This case highlights the importance of maintaining a broad differential diagnosis for non-healing ulcers and employing a methodical approach to arrive at a definitive diagnosis.

## Case presentation

**Figure 1:**
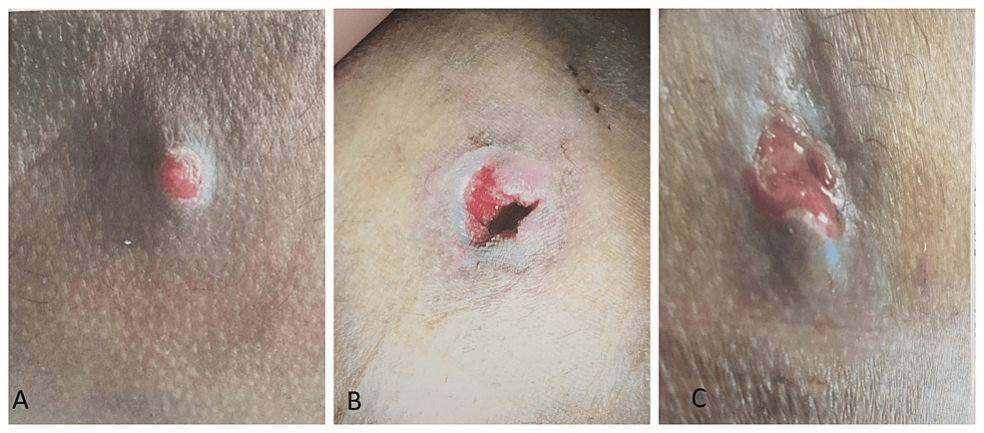
Presentation and progression of a skin lesion A: Lesion around 20 mm in diameter (initial presentation) B: Lesion after incision and drainage C: Lesion draining copious amounts of clear fluid after one week of incision and drainage

A 39-year-old Indian male has developed a small abscess of 20 mm in diameter on his right gluteal region for the past two weeks (Figure 1A). Past history includes cigarette smoking for 10 pack years and quitting smoking 10 years ago. There was also a history of hypertension for the last four years, with moderate derangement of the lipid profile for the last three years (Table 1) [14]. There is no previous history of similar lesions. There is no family history of Crohn's disease. No mucosal ulcers are present. There is no history of chest pain or pain in the extremities. There is no history of loss of appetite. There is no history of weight loss. There is no history of weakness. There was no sensory loss over the lesion. 

Incision and drainage were done, which yielded pus, and was treated with antibiotics (Amoxicillin 500 mg with Clavulanic acid 125 mg) for five days (Figure 1B). The patient presented again after two weeks as the lesion failed to resolve, and now the lesion had increased in size, appearing to be like a pyogenic granuloma. A pus culture was performed, which yielded a coagulase-negative *Staphylococcus* organism. Thus again, incision and drainage were done, and the antibiotic (Cefixime 250 mg) was prescribed for 10 days as per the culture and sensitivity report. 

The patient returned after four weeks with complaints of new-onset painful right inguinal swelling and no improvement in the lesion (Figure 1C). On examination, clear fluid was oozing out from the wound, and the presence of right inguinal lymphadenopathy was noted, which was firm in consistency and measured 50 mm in diameter. No other lymph nodes were enlarged. 

This raised the immediate need to perform a biopsy to detect a possible underlying cause. Thus, a biopsy was performed, which showed atypical hematopoietic cells with dispersed chromatin and multiple nucleoli; thus, the diagnosis of lymphoma became certain with the help of immunohistochemistry and biopsy results after eight weeks of the patient noticing an ulcer. The fine needle aspiration cytology (FNAC) report further consolidated the diagnosis.

**Figure 2:**
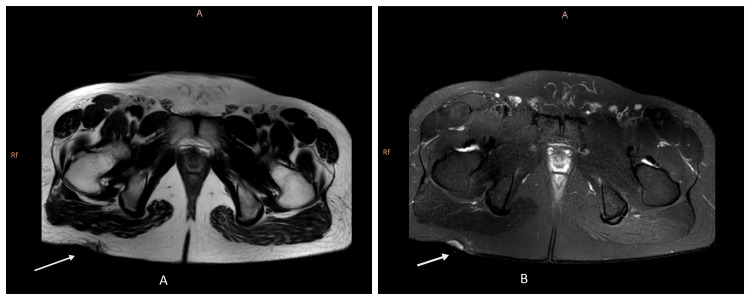
MRI gluteal region axial view A and B indicate a heterogenous signal intensity lesion seen in the subcutaneous plane in the right gluteal region, measuring approximately 21 x 8 x 21 mm. Underlying muscles are normal. No extension was seen into the pelvic cavity or underlying bones. Underlying neurovascular bundles and sciatic nerves are normal. No abnormal flow void was seen within the lesion. Multiple small inguinal lymph nodes are seen. Suggestive of mitotic pathology.

A diagnostic workup revealed abnormalities in several areas. A complete blood count showed mild lymphocytosis and eosinophilia, but the white blood cell count was within normal limits (Table 1). The renal function tests, along with the liver function tests, yielded normal results (Tables 2, 3). A biopsy of the ulcer confirmed a malignant neoplasm, likely originating from hematopoietic tissue with dispersed chromatin and multiple nucleoli with a background of mixed neutrophils, plasma cells, small lymphocytes, and histiocytes, along with tissue edema and congestion. Immunohistochemistry testing was strongly positive for CD30, focally positive for CD45, and negative for other markers. Further examination of the right inguinal lymph node via FNAC identified atypical lymphoid cells in various stages of development with scattered neutrophils, plasma cells, histiocytes, multinucleated cells, and crushed nuclear material, suggestive of lymphoid malignancy. An ultrasound of the gluteal region identified a fluid collection within the subcutaneous tissue, consistent with an abscess. Finally, MRI imaging revealed a heterogeneous signal-intensity lesion measuring approximately 21 x 8 x 21 mm in the subcutaneous plane of the right gluteal region. Additionally, multiple small lymph nodes were observed in the inguinal region (Figure 2) [15] Table 4 shows miscellaneous information. 

**Table 1:** Hematology results WBC: White blood cells, RBC: Red blood cells, MCV: Mean corpuscular volume, MCH: Mean corpuscular hemoglobin, MCHC: Mean corpuscular hemoglobin concentration, RDW: Red cell distribution width, ESR: Erythrocyte sedimentation rate, FBS: Fasting blood sugar, HbA1c: Glycosylated hemoglobin, HDL: High-density lipoprotein, LDL: Low-density lipoprotein, TG: Triglycerides

Hematology	Results	Reference Range
WBC count (1000/cumm)	8.8	4-11
Platelet count (1000/cumm)	253	150-450
RBC count (million/ul)	4.73	4.5-5.1
Hemoglobin (gm/dl)	14.3	13.5-17.5
MCV (fl)	90.1	80-100
MCH (pg)	30.2	27.5-33.2
MCHC (gm/dl)	33.2	33.4-35.5
RDW (%)	12.6	11.6-14.0
Neutrophills (%)	78	40-80
Neutrophill absolute count (/cumm)	4488	1700-7000
Lymphoytes (%)	14	20-40
Lymphocytes absolute count (/cumm)	3124*	900-2900
Eosinophills (%)	04	1-6
Eosinophills absolute count (/cumm)	546*	50-500
Monocytes (%)	01	2-10
Basophills absolute count (/cumm)	44	0-300
Basophills (%)	00	0-1
ESR (mm/hr)	20	0-20
FBS (fasting blood sugar) (mg/dl)	120*	60-100
HbA1c (glycosylated hemoglobin) (%)	5.7	Less than 5.7
HDL (high-density lipoprotein) (mg/dl)	50*	More than 60
LDL (low-density lipoprotein) (mg/dl)	158*	Less than 100
TG (triglycerides) (mg/dl)	210*	Less than 150

**Table 2:** Renal function test

Renal Function test	Results	Reference Range
Glucose (mmol/L)	5.81	3.9-7.7
Urea (BUN) (mmol/L)	3.5	1.7-8.3
Creatinine (umole/L)	67.65	61.8-114.9
Sodium (mmol/L)	136.3	136.0-145.0
Potassium (K) (mmol/L)	4.62	3.5-5.1
Inorganic Phosphate (P) (mmol/L)	1.41	0.8-1.45

**Table 3:** Liver function test ALP: Alkaline phosphatase, ALT (SGPT): Alanine aminotransferase, AST (SGOT): Aspartate aminotransferase

Liver Function Test	Results	Reference Range
Albumin (g/L)	43.0	35.0-52.0
Gama glutamyl transferase (GGT) (U/L)	44.0	0.0-55.0
ALP (U/L)	95.0	53.0-128.0
ALT (SGPT) (U/L)	58.3*	0.0-45.0
AST (SGOT) (U/L)	31.4	0.0-35.0
Total bilirubin (umole/1)	6.58	0.0-33.9
Direct bilirubin (umole/l)	3.31	0.0-5.1
Total protein (g/L)	74.7	64.0-83.0

**Table 4:** Miscellaneous

Miscellaneous	Results	Reference Range
C- Reactive Protein [mg/dl]	1.44	0-5
Lactogen Dehydrogenase [ U/L]	448	225-450

Treatment

After two failed incisions and drainage, along with courses of antibiotics (Amoxicillin 500 mg with Clavulanic acid 125 mg and later Cefixime 250 mg), the finding of atypical cells on biopsy along with immunohistochemistry positive for CD30 and focally positive for CD45 raised suspicion of lymphoma. The FNAC of the right inguinal lymph node also indicated the presence of atypical cells. Thus, chemotherapy was started with Brentuximab.

Brentuximab was administered in 16 cycles at an interval of 21 days. The first two doses of 1.6 mg/kg body weight were followed by 1.4 mg/kg body weight, as derangement of liver enzymes was observed after two doses [16-18].

The lesion healed considerably, along with a dramatic reduction in the size of the inguinal lymphadenopathy, after just one cycle of Brentuximab. The patient tolerated chemotherapy well, and the lesion also healed completely after five months of chemotherapy. The results of regular follow-up for recurrence every six months have been negative for the last 18 months.

## Discussion

Maintaining a broad differential diagnosis was crucial in this case. Given the initial presentation, a bacterial infection was the most likely culprit. Treatments targeting *Staphylococcus aureus* and *Pseudomonas aeruginosa* were attempted [1] but proved ineffective, ruling out a common bacterial cause. Fungal infections, though less frequent, were also considered [2] but ruled out given the initial bacterial growth on pus culture and complete blood profile. The negative interferon-gamma release assay argued against tuberculosis [3]. Hansen’s disease was also primarily ruled out with a pin-prick test and clinical presentation [19].

Vascular insufficiency [4] and autoimmune disorders [5] were other possibilities explored. However, the patient's medical history included risk factors like a smoking history of 10 pack years and hypertension for the last four years, with moderate derangement of the lipid profile for the last three years, which contributed to suspicion of compromised circulation or autoimmune conditions [14]. However, a lack of history of coronary artery disease or discoloration or claudication in extremities ruled out the possibility. Additionally, the ulcer's characteristics and treatment response did not align with these etiologies.

Malignancy, although less common than the infectious etiology for non-healing ulcers, eventually emerged as the leading suspect. The persistent nature of the ulcer despite interventions and the atypical cells identified in the biopsy prompted further investigation. While the initial workup couldn't definitively subtype the lymphoma, the positive CD30 [18] and focal CD45 expression on immunohistochemistry pointed towards a T-cell lymphoma [16,17]. This case highlights the importance of considering malignancy in patients with non-healing ulcers and employing a stepwise approach that includes a biopsy for a definitive diagnosis.

## Conclusions

This case presentation underscores the complexities of diagnosing non-healing ulcers. The initial masquerade as a bacterial infection highlights the necessity for a multifaceted differential diagnosis. While conventional treatments for bacterial etiologies proved futile, the persistent ulcer ultimately unmasked a cutaneous anaplastic T-cell lymphoma. This case serves as a powerful reminder to maintain a high index of suspicion for malignancy in patients with non-healing wounds. The crucial role of biopsy and FNAC in achieving a definitive diagnosis is further emphasized. The patient's considerable response to Brentuximab further consolidates the results of studies done on this drug and prevents the sequelae of untreated anaplastic T-cell lymphoma, i.e., wide-spread nodal and visceral disease. This case stands as a testament to the imperative of clinical vigilance and a meticulous diagnostic approach, ultimately leading to the unmasking of a disguised malignancy and the potential for improved patient outcomes.
